# Estimating Underdetection of Foodborne Disease Outbreaks (Response)

**DOI:** 10.3201/eid3011.241351

**Published:** 2024-11

**Authors:** Laura Ford, Julie L. Self, Karen K. Wong, Robert M. Hoekstra, Robert V. Tauxe, Erica Billig Rose, Beau B. Bruce

**Affiliations:** Centers for Disease Control and Prevention, Atlanta, Georgia, USA

**Keywords:** Foodborne outbreaks, bacteria, outbreak size, power law

**In Response:** Our analysis of foodborne outbreaks reported in the United States demonstrated that foodborne outbreaks are distributed approximately according to a family of probability distributions—that is, power laws—based on our analysis of culture-confirmed cases in a national surveillance system (*1*). Hedberg et al. suggest restricting analyses by common detection pathways, hypothesizing that distinct pathways may follow distinct power law distributions (*2*). That is a notable idea based on real surveillance concerns, but it is challenging to explore in the available data. Analyses of the distribution of events, which vary by many orders of magnitude, require large amounts of data because identifying the underlying distribution is primarily based on the distribution of the rarer events in the tails of the distribution. Furthermore, the detection pathway is not generally contained in the surveillance record.

However, the reported estimated case count is available from the surveillance record and likely captures aspects of the distinct pathways noted by Hedberg et al. Notable variation exists in their reporting over time and by jurisdiction and is, in part, why we did not choose estimated cases for our primary analysis. We did explore the power law fit of 2 very common pathogens, *Salmonella* and norovirus, which Hedberg et al. noted are likely to have different pathways for how cases are reported to surveillance. The fit of confirmed *Salmonella* cases and estimated norovirus cases appear consistent with approximate power law distributions (Figure): *Salmonella* Kolmogorov-Smirnov statistic (KS) = 0.028, p = 0.607; norovirus KS = 0.036, p = 0.437. The fit of estimated cases overall also appears consistent with an approximate power law distribution (KS = 0.026, p = 0.191; minimum threshold 80, 90% credible interval 49–117; slope 2.64, 90% credible interval 2.50–2.79), which further supports the validity of power laws as descriptors of outbreak size, regardless of the underlying mechanism of discovery and reporting.

**Figure:**
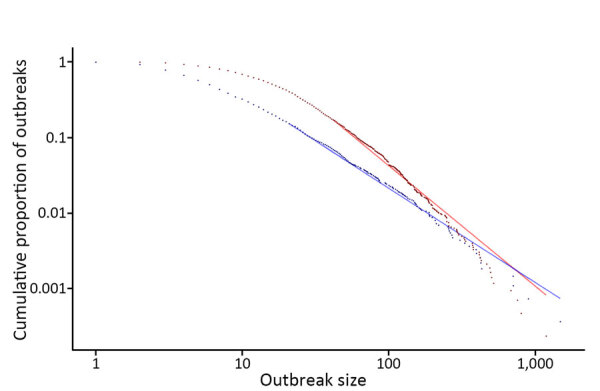
Log-log scale of foodborne *Salmonella* and norovirus outbreak size versus frequency from a power law for estimating underdetection of foodborne outbreaks, United States. Actual *Salmonella* (blue points) versus expected *Salmonella* (blue line) using laboratory-confirmed cases (minimum threshold 21, 90% credible interval [CrI] 11–43; slope 2.2, 90% CrI 2.1–2.5) and actual norovirus (red points) versus expected norovirus (red line) using estimated cases (minimum threshold 42, 90% CrI 22–123; slope 2.6, 90% CrI 2.3–3.3).
